# Inflammation in glomerular diseases

**DOI:** 10.3389/fimmu.2025.1526285

**Published:** 2025-03-04

**Authors:** Yongqing Xiong, Wei Li, Songzhi Jin, Shujing Wan, Suzhen Wu

**Affiliations:** ^1^ School of Rehabilitation Medicine, Gannan Medical University, Ganzhou, China; ^2^ School of Basic Medicine, Gannan Medical University, Ganzhou, China; ^3^ Key Laboratory of Prevention and Treatment of Cardiovascular and Cerebrovascular Diseases, Ministry of Education, Gannan Medical University, Ganzhou, China

**Keywords:** glomerular diseases, inflammation, cytokines, glomerular cells, signaling pathway

## Abstract

The structural and functional integrity of glomerular cells is critical for maintaining normal kidney function. Glomerular diseases, which involve chronic histological damage to the kidney, are related to injury to glomerular cells such as endothelial cells, mesangial cells (MCs), and podocytes. When faced with pathogenic conditions, these cells release pro-inflammatory cytokines such as chemokines, inflammatory factors, and adhesion factors. These substances interact with glomerular cells through specific inflammatory pathways, resulting in damage to the structure and function of the glomeruli, ultimately causing glomerular disease. Although the role of inflammation in chronic kidney diseases is well known, the specific molecular pathways that result in glomerular diseases remain largely unclear. For a long time, it has been believed that only immune cells can secrete inflammatory factors. Therefore, targeted therapies against immune cells were considered the first choice for treating inflammation in glomerular disease. However, emerging research indicates that non-immune cells such as glomerular endothelial cells, MCs, and podocytes can also play a role in renal inflammation by releasing inflammatory factors. Similarly, targeted therapies against glomerular cells should be considered. This review aims to uncover glomerular diseases related to inflammation and pathways in glomerular inflammation, and for the first time summarized that non-immune cells in the glomerulus can participate in glomerular inflammatory damage by secreting inflammatory factors, providing valuable references for future strategies to prevent and treat glomerular diseases. More importantly, we emphasized targeted glomerular cell therapy, which may be a key direction for the future treatment of glomerular diseases.

## 1 Introduction

Glomerular disease (GD) is divided into two main categories: primary glomerular disease and secondary glomerular disease. The former mainly includes focal segmental glomerulosclerosis (FSGS), minimal change disease (MCD), IgA nephropathy (IgAN), primary membranous nephropathy (PMN), membranoproliferative glomerulonephritis (MPGN), and primary crescentic glomerulonephritis, while the latter includes lupus nephritis (LN), HIV-associated nephropathy (HIVAN), diabetic nephropathy (DN), hypertensive nephrosclerosis, post-infectious glomerulonephritis (PIGN), non-IgA membranoproliferative glomerulonephritis (MesPGN), and hypo-immunoglobular nephritis (1–4). These glomerular diseases result from glomerular injury, severe tubular loss or atrophy, some cystic degeneration, and thickening of the renal vasculature, are considered to be one of the major causes of progressive end-stage renal disease. The glomerular disease can be definitively diagnosed by renal biopsy, histopathologic examination, and laboratory tests. Several studies have shown a multimodal progression of glomerular diseases, which is mainly characterized by inconsistent prevalence of different glomerular diseases in different regions. Focal segmental glomerulosclerosis (FSGS; 19.1%) predominated in North America; lupus nephritis (38.1%) and FSGS (15.8%) predominated in Latin America; IgA nephropathy (IgAN; 22.1%) and FSGS (14.9%) predominated in Europe; and IgAN (39.5%) and lupus nephritis (16.8%) predominated in Asia (5). In China, MCD is the most common glomerular disease, accounting for 28.7% of primary glomerular diseases, while MPGN and IgAN account for 25.8% and 22.1%, respectively, in the second and third places (6). It is safe to assume that LN and DN are the most common secondary glomerular diseases and this finding is prevalent worldwide (2, 7). Despite the fact that the occurrence of glomerular disease varies globally (1), it is known to have an impact on mortality rates associated with chronic kidney disease (8, 9).

The glomerular filtration barrier is composed of three main components: glomerular endothelial cells, MCs and podocytes. Investigating the impairment of those components is a major focus in the study of glomerular diseases. Glomerular endothelial cells have a similar role to other endothelial cells, help maintain tissue perfusion and hemodynamics by constructing a filtration barrier, and regulating the inflammatory process through the expression or binding of various circulating factors, such as endothelin-1 (ET-1), prostacyclin inflammatory receptor, intercellular adhesion molecule 1 (ICAM1), vascular cell adhesion protein 1 (VCAM1), E-selectin and membrane cofactor protein 1 (MCP1) among others (10–12). Mohamed A demonstrated that intravenous injection of ET-1 into the glomerulus caused an increase in plasma soluble intercellular adhesion molecule-1 (sICAM-1) and MCP-1 in the glomerulus, as well as an increased in the number of macrophages and lymphocytes in the renal cortex (13). Studies have demonstrated that overexpression of ET-1 in the kidney leads to renal inflammation and fibrosis (14). When glomerular cells are injured, they stimulate the secretion of inflammatory factors. As a kind of stromal cell, in addition to organizing the glomerular structure and maintaining the homeostasis of endothelial cells and podocytes, MCs also participate in the immune response through the production of chemokines (including Col6a3, Mmp14, Mmp17, Col12a1, Cxcl1, Ccl2 and Cx3cl1) (15, 16), which provides the basis for the development of glomerular inflammation. The chemokines Ccl2 and Cx3cl1 have also been found to be involved in the development of inflammation in DN (17). Glomerular disease progression is often linked to inflammation caused by podocyte injury. Inflammatory factors, primarily originating from podocyte injury, are commonly overexpressed in various glomerular diseases. In an experiment by Rachael D. Wright et al., podocytes were exposed to inflammatory factors (IL-1β, TNF-α, IFN-α, and IFN-γ) individually or in combination. The aim was to establish an *in vitro* renal inflammation model that mirrors the inflammatory conditions observed in patients with lupus nephritis (LN). As a result of this stimulation, there was a rise in the secretion of IL-6, IL-8, IL-10, VEGF, M-CSF, and interferon gamma-inducible protein-10 (IP-10) (18).

Inflammatory factors released after glomerular cell injury may play a crucial role in the progression of glomerular diseases. Studies have shown that oxidative stress can also trigger the release of inflammatory factors from glomerular cells (19, 20). ROS overproduction and impaired antioxidant defense systems are the two leading causes of oxidative stress, which is an imbalance between ROS production and elimination (21) This imbalance is often seen in mitochondria, where excess ROS production can be harmful and cause further damage to mitochondria. This can lead to elevated mtROS levels, reduced ATP production, and ultimately result in mitochondrial metabolic disorders (22–24). Increased levels of ROS in renal tubular epithelial cells can induce the secretion of chemokines and pro-inflammatory cytokines, such as interleukin-1β (IL-1β), through NOD-like receptors (NLRs), leading to sustained kidney injury (25). High glucose levels induce ROS in glomerular mesangial cells, activating the NF-KB pathway and increasing expression of EGR-1 and PKC-α in MCs of patients with DKD. This activation enhances inflammatory factors MCP-1 and fibrotic markers collagen I and III, promoting localized inflammatory and fibrotic responses in the kidney (26). In glomerular endothelial cells, elevated glucose levels increase the expression of insulin-like growth factor-binding protein 5 (IGFBP5) in endothelial cells, leading to upregulation of EGR1. In addition, it was found that EGR1 enhances the enzymatic function of PFKB3, which leads to the secretion of inflammatory factors such as ICAM-1, TNF-α, IL-6, and MCP-1 from endothelial cells through the enhancement of glycolysis, and ultimately causes inflammatory injury (27). These effects are linked to disruptions in mitochondrial metabolism. Furthermore, when mtROS induce mitochondrial permeability transition pores (mPTP), IMM proteins, such as cytochrome c, are released into the cytosol, resulting in inflammatory response and apoptosis (28).

Inflammatory response caused by glomerular cell injury is a key factor in the progression of glomerular diseases and increase the risk of cardiovascular and all-cause mortality. While immune cells have long been known to be involved in the development of inflammation, recent studies have shown that non-immune cells such as glomerular endothelial cells, mesangial cells (MCs), and podocytes could also contribute to renal inflammation by releasing inflammatory factors. This article provides a review of research on the molecular mechanisms, prevention, and treatment of inflammatory responses in various types of glomerular diseases. Understanding the signaling pathways involved in triggering glomerular diseases in pathological conditions and implementing strategies to prevent inflammatory responses can help protect against glomerular damage.

## 2 Structure and function of the glomerulus

**Figure 1:**
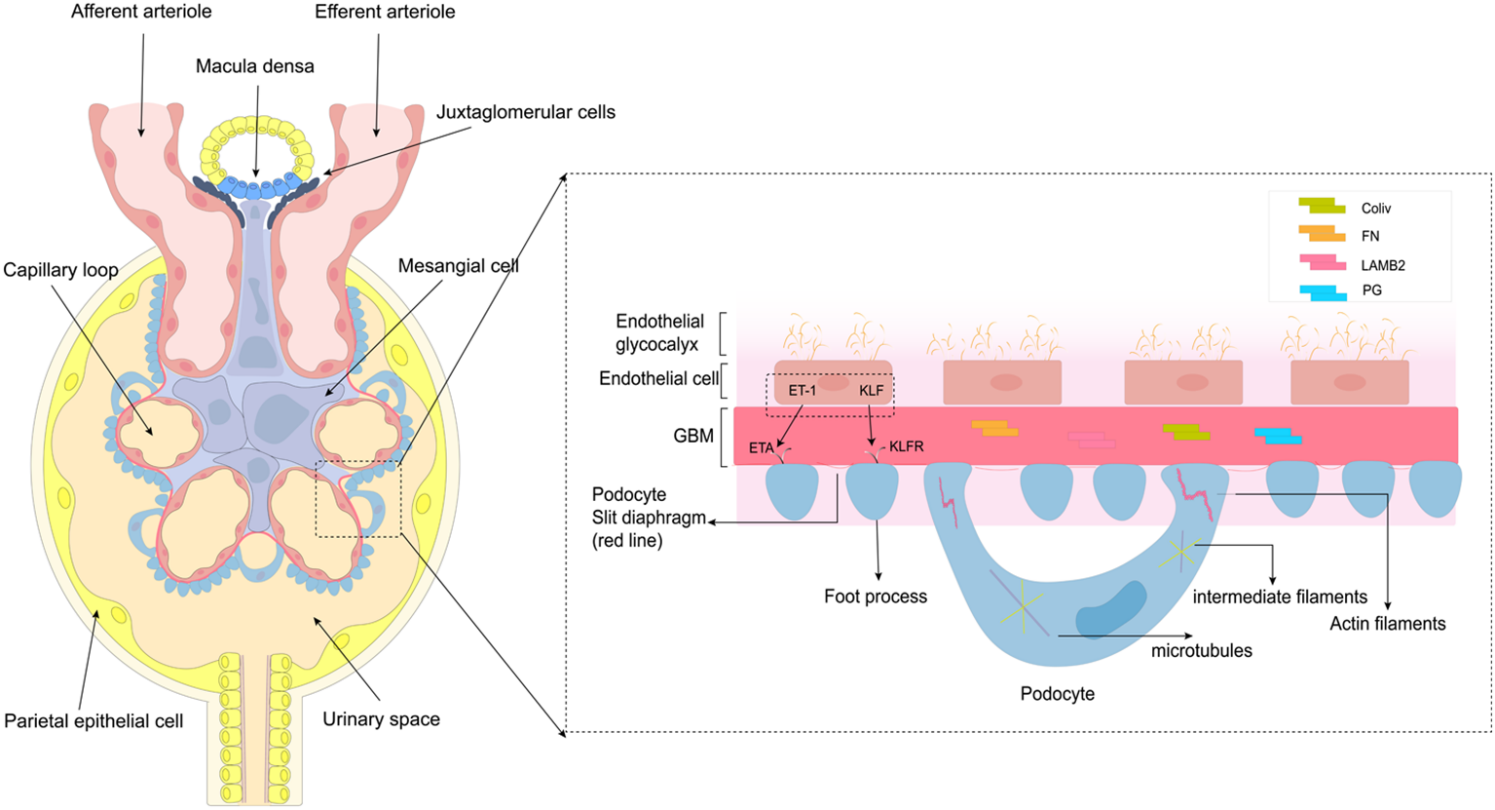
Structure and function of the glomerulus. This diagram depicts the structure and function of glomerular endothelial cells, glomerular basement membrane, and podocytes, primarily podocytes. Podocytes consist of cell bodies, primary processes, and secondary foot processes; adjacent foot processes form the slit diaphragm. The cell bodies and primary processes are based on microtubules and intermediate filaments, while the foot processes mainly depend on the actin filaments.

The glomerulus is a filtration system consisting of a central capillary bulb and a renal capsule that filters blood and forms primary urine. It consists of an open endothelium layer of endothelial cells, a glomerular basement membrane (GBM) made up of extracellular proteins in the second layer; and the distal layer consists of visceral epithelial. Podocytes contribute to the formation of filtration slit septa, and play a key role in supporting capillary flow and maintaining the integrity of the freestanding capillary loop (29). Glomerular disease can occur due to damage to any of the structures in the glomerular filtration barrier, leading to inflammation. The three types of glomerular cells, including the MCs, have endocrine functions and interact with each other through autocrine and paracrine pathways, which can contribute to the inflammation in the glomerulus (**Figure 1**).

### 2.1 Endothelial cell

Glomerular endothelial cells (GEnC) are a vital part of the glomerulus, responsible for maintaining the filtration barrier. These cells are attached to the inner glomerular basement membrane, and are separated from the podocytes by the basement membrane. The open window, found in the cytoplasmic portion of the cytosol, opposite the podocyte pedicle, and passes through the filtration slit of the glomerular basement membrane. This structure not only plays a key role in glomerular permeability but also facilitates communication between GEnC and neighboring cells (30). The glycocalyx, a specialized structure found in GEnC (31), plays a crucial role in limiting the penetration of macromolecular proteins (32). Notably, there is growing evidence that loss of endothelial openings and a decrease in the number of glycocalyx are among the earliest changes in glomerular disease (33), and is also associated with inflammation. A study revealed that in patients with type 2 diabetes mellitus, serum levels of tumor necrosis factor (TNF) receptors 1 and 2 were inversely related to the percentage of open-window GEnC (34). Moreover, dysfunction in GEnC opening and a decrease in glycocalyx quantity can hinder the interaction between GEnC and adjacent glomerular cells, leading to irregular expression of certain cytokines (30, 32). There is evidence that ET-1 and Krüppel-like factor (KLF) mediate communication between endothelial cells and podocytes and bind to corresponding receptors to promote/inhibit inflammation (13, 35). In DN, ET(A) receptor-induced oxidative stress in endothelial mitochondria has been shown to lead to GEnC damage and loss of open windows (36). Mitochondrial oxidative stress induces GEnC dysfunction, causing podocyte injury (37). KLF acts as a transcription factor that inhibits NF-KB activation, resulting in anti-inflammatory effects (38, 39). Endothelial openings and glycocalyx play a key role in the glomerular cell communication and the development of glomerular inflammation.

### 2.2 Mesangial cells

Mesangial cells (MCs), as resident cells of the kidneys, play an important role in maintaining glomerular function. They are located between the capillary loops of the glomeruli and are adjacent to endothelial cells and the capillary basement membrane (40). Mesangial cells are now recognized to belong in a class of cells known as mesenchymal stromal cells. Consistent with their role as stromal cells, MCs are the main producers of glomerular matrix, which comprises type iv collagen (Col iv), fibronectin (FN), laminin subunit beta 2 (LAMB2), proteoglycans (PG) and other ECM components (41, 42). The main functions of MCs include providing support to the capillary plexus, constricting vascular and secretion of extracellular matrix components, producing cytokines such as Col6a3, Mmp14, and Mmp17, as well as chemokines like Ccl2 and Cx3cl1, and carrying out phagocytosis and clearance of macromolecules (15). MCs can be affected by changes in the glomerular environment, leading to functional transformation. They can act as stromal cells and influence the recruitment of immune cells by altering ECM components and producing chemokines. This can result in a quick response to innate immune stimuli or tissue damage. MCs also have the potential to function as antigen-presenting cells, expressing surface markers like MHC and ICAM-1 when activated. They can interact with CD4 T cells to participate in the local renal immune response (43). Additionally, studies have shown that mast cells (MCs) also express innate immune signaling components such as Toll-like receptors TLR3 and TLR4, as well as intracellular pattern recognition receptors NOD1 and NOD2 (16). In summary, the interaction between MCs and the immune system leads to the production of various chemokines and matrix components that regulate the inflammatory response, aiding in the characterization of inflammation in glomerular disease.

### 2.3 Podocytes

Podocytes, the glomerular basement membrane (GBM), and endothelial cells make up the glomerular filtration barrier (GFB) (44). The foot process of podocytes can be divided into three specialized membrane regions: the basal, apical, and lacunar septum regions. The apical membrane region plays a crucial role in the negatively charged glomerular barrier; with the slit diaphragm regulating glomerular permeability. In addition, podocytes have a well-organized cytoskeletal structure that includes microtubules (MTs) and intermediate filaments (IFs). It is crucial for maintaining GFB integrity and regulating cell structure, stability, motility, cell adhesion, and slit diaphragm insertion (45). Sijia Ma et al. emphasized the importance of foot cytoskeletal rearrangements in glomerular diseases. The slit diaphragm interacts with various focal adhesion proteins, causing the breakdown of the foot cytoskeletal structure. This includes the involvement of signaling node nephrin, calcium influx through transient receptor potential channel 6 (TRPC6), and regulation of the Rho family, ultimately resulting in the disruption of the initial cytoskeletal framework (46). The slit diaphragm also contains lipid rafts that are abundant in sphingolipids, which serve a structural purpose in cell membranes and possess various bioactive functions (47). Dysregulation in sphingolipid metabolism has been shown to cause podocyte injury and drive the progression of glomerular disease (48). Podocytes, as the critical component of the kidney filtration units, maintain their unique cellular structure through an intricate and coordinated network of cytoskeletons (49). When podocytes suffer direct or indirect injury, it disrupts the cytoskeleton, leading to foot process effacement (FPE), proteinuria, and ultimately kidney inflammation (46, 50).

## 3 Inflammatory factors in glomerular disease

### 3.1 Three types of glomerular cells and inflammatory factors

Inflammation plays a significant role in the onset of chronic kidney disease, leading to reduced renal blood flow and glomerular filtration rate (GFR) (51). Glomerular endothelial cells, mesangial cells, and podocytes, as resident cells, function collectively to preserve the structure and function of the glomerulus, and establish communication with one another. When inflammation occurs, renal resident cells become activated and display a pro-inflammatory phenotype (10, 52), this activation involves the release of transcription factors, pro-inflammatory factors, chemokines, and adhesion factors, ultimately resulting in glomerular injury and fibrosis (53, 54). Immune injury, metabolic injury, toxicity, and genetic injury can all lead to the activation of glomerular cells (16). Immune injury is mainly characterized by macrophage recruitment and upregulation of some cytokines, including inflammatory factors, adhesion factors, and chemokines (16, 55). Under these pathological circumstances, glomerular cells crosstalk with each other via signaling and inflammatory mediators, ultimately resulting in glomerular damage (54, 56, 57). In summary, the activation of these three kinds of glomerular cells in pathological conditions leads to the release of pro-inflammatory factors, exacerbating glomerular injury and ultimately causing glomerular disease.

It has been reported that all three types of glomerular cells can express inflammatory factors (**Table 1**) and lead to glomerular diseases. Here, we will explore the pathophysiological processes of these four glomerular diseases, which are triggered by glomerular cell injury and the inflammatory response.

**Table 1:** Inflammatory molecules associated with glomerular disease.

Glomerular cells	Category	Molecules
Endothelial cells	Transcription factors	KLF (58), NF-KB (59)
	Pro-inflammatory cytokines	IL-6 (60), IL-1 (61), TNF-α (62), IFN-α (63)
	Chemokines	MCP-1(Ccl2) (64)
	Adhesion molecules	ICAM1 (65), VCAM1 (66), E- selectin (67)
	other	ET-1 (68), IGFBP5 (27)
Mesangial cells	Transcription factors	NF-KB (69, 70)
	Pro-inflammatory cytokines	IL-6 (71, 72), IL-8 (73), TNF-α (74)
	Chemokines	Col6a3 (75), Mmp14, Mmp17, Col12a1, CXCL10 (75), MCP-1(CCL2) (76), CCL5 (75),Cx3cl1 (77),
	Adhesion molecules	ICAM1 (78), α-Actinin 4 (79)
	other	MIF (80)
Podocytes	Transcription factors	KLF (58), NF-KB (81)
	Pro-inflammatory cytokines	IL-6 (82), IL-8 (83), IL-10, TNF-α (84)
	Chemokines	CXCL1, CCL2 (85),
	Adhesion molecules	α-Actinin 4 (86)

### 3.2 Glomerular injury resulting from inflammation

#### 3.2.1 Inflammation associated with diabetic nephropathy can cause damage to the glomeruli

DN is a progressive microvascular diabetic complication and one of the major causes of end-stage renal disease (ESRD), which is clinically characterized by persistent hyper-proteinuria and reduced glomerular filtration (87). Recently, numerous studies have shown that DN may be characterized as a chronic inflammatory kidney disease, with the upregulation of inflammatory signaling pathways and the infiltration of inflammatory cells are associated with kidney injury and the development of DN (88, 89). Reviews have indicated that several inflammatory factors are involved in DN inflammatory processes, such as nuclear transcription factors (NF-κB), pro-inflammatory cytokines (IL-1, IL-6, IL-18 and TNF), chemokines (MCP-1, CXCL12, CX3CL1 and CX3CR1) adhesion molecules (ICAM1, VCAM1, E-selectin and α-actinin 4), and signaling molecules (STAT1,STAT3 and STAT5) are involved in DN inflammatory processes (17, 90). Yuheng Qiu et al. identified the biological pathways involved in the development of DN by enrichment analysis, revealing that chemokines, cytokines, and inflammation-related pathways were strongly associated with the progression of DN to end-stage renal disease (91). Evidence demonstrated that TNF-α, a pro-inflammatory factor, is necessary for the development of DN (92).

Ioanna et al. found evidence indicating that the TNF-α pathway is activated in the early phases of DN. They observed elevated levels of TNF-α in the urine of patients with early DN compared to those in later stages (93). Elevated levels of TNF-α receptors (TNFR1 and TNFR2) and the kidney injury marker KIM-1 have also been linked to DN (94), TNFR1 and TNFR2 have been recognized as markers for the risk of developing ESRD in people with type 2 diabetes (95). TNF-α is an inflammatory mediator that binds to TNFR1 and TNFR2, leading to the activation of various signaling pathways. This results in the expression of transcription factors, cytokines, growth factors, receptors, cell adhesion molecules, and other inflammatory mediators. When bound to TNFR2, TNF-α, a downstream target of the NF-KB pathway, induces persistent NF-κB activation. It also triggers MCs by releasing chemokines like MCP-1, IL-6, and IL-8 (96),while directly promotes the expression of ICAM-1 on MCs (97). In summary, TNF-α is responsible for initiating and development of DN inflammation, mainly by stimulating the production of adhesion molecules and releasing chemokines when it binds to TNFR. Interestingly, MCP-1 and TNF-α are recognized as novel inflammatory biomarkers for the diagnosis of DN (98).

#### 3.2.2 Lupus nephritis

Systemic lupus erythematosus that affects the kidneys is commonly referred to as LN, which is a serious and frequent complication. Various models have been studied to uncover the cause of LN, and most of these modern models suggest that anti-chromatin antibodies have a significant impact on the initiation of LN, mainly by binding to exposed chromatin in the glomerulus. In the early stages of LN, there is often an accumulation of chromatin fragments and IgG complexes in the mesangial matrix, likely due to the decrease in DNase I enzyme activity. Therefore, the failure to degrade chromatin from secondary necrotic cells leads to its accumulation with IgG complexes in the GBM (99–102). Since the mesangial membrane is one of the major sites of IG deposition, the development of LN inflammation may be associated with MCs (103). It has been demonstrated that anti-dsDNA antibody binds directly to MC membrane connexin II and α-actinin to induce the expression of pro-inflammatory factors in cultured MCs, including TNF-α, IL-1β, IL-6 (104, 105). A recent study has indicated that IL-6, a well-known pro-inflammatory factor, does not appear to be linked to the emergence of LN (106). Anti-dsDNA antibody also induces MCP-1 secretion and CXCL1 and CX3CL gene expression in cultured MCs through activation of PKC, increased IL-1β secretion, and IκB and NFκB signaling pathways (105, 107, 108). The MCs possess TLR receptors, and upon activation, these receptors initiate pathways that lead to the generation of various adhesion molecules, cytokines, and chemokines (109). Moreover, in the LN mouse model, the adhesion molecules ICAM-1 and VCAM-1 showed an increase in expression in MCs (110). A recent study has shown that the activation of NLRP3 in podocytes may be a contributing factor to LN (111). Moreover, the expression of proinflammatory cytokines (such as IL-1 and IL-6) by classically activated macrophages leads to the proliferation of MCs and the expression of extracellular matrix (112). MCs contribute significantly to IL-6 production in glomeruli and also releases M-CSF, which triggers the recruitment of macrophages in glomeruli (113). Thus, it is evident that macrophages are also involved in the development of LN inflammation. A recent study proposed that macrophages may be a marker for the onset and remission of inflammation in LN (114). Traditional markers for detecting LN, including serum creatinine, urinary protein, anti-dsDNA antibody, and complement C3/4, do not directly reflect the onset of LN or differentiate between active and chronic disease. Some studies have reported that rinary CD11c+ macrophages derived from circulating monocytes are abundant in the urine of patients with active proliferative LN and are significantly associated with the serum anti-dsdsorbutin antibody. significantly associated with the serum anti-dsDNA antibody titer, inflammation and interstitial fibrosis (115). Soluble CD163 is the most discussed macrophage product, and it can be detected in the urine of LN patients (116). Moreover, some studies have reported that patients with active LN have significantly higher levels of urinary soluble CD163 (117). In summary, CD11c+ and CD163 can be used as biomarkers for clinical and pathological features of LN patients (114, 116).

#### 3.2.3 Focal segmental glomerulosclerosis

FSGS is characterized by the formation of glomerular scarring and capillary occlusion in specific glomerular clusters due to extracellular matrix deposition (118). Studies have indicated that inflammation can exacerbate glomerulosclerosis and progress to end-stage renal disease. Recently, there have been several new pathogenic mechanisms proposed, such as circulating permeability factors that are able to activate inflammatory factors in glomeruli and subsequently induce glomerulosclerosis (119). Lilian Otalora et al. performed gene-enriched KEGG pathway analysis of glomeruli from FSGS patients and showed that inflammatory pathways including TNF-α, IL-17, and NF-κB were significantly activated by one or more circulating permeability factors (120). Furthermore, they also showed that chemokines such as CCL2, CCL3, CCL20, CXCL1, CXCL2, CXCL5, CXCL6, and CXCL12 were activated in the podocytes of FSGS patients. Notably, CCL3 was only rapidly activated when it was exposure to circulating factors present in the serum of FSGS patients (120). Another study revealed that CCL2/CCR2 signaling might contribute to the damage of glomeruli in FSGS (85). The absence of CCL2 has been linked to a reduction in both structural and functional damage to the kidney in glomerulosclerosis. Moreover, individuals with primary FSGS have shown elevated levels of IL-9. Experiments have shown that blocking the attachment of the IL-9 receptor to podocyte membranes can effectively prevent glomerulosclerosis (121). These cytokines not only reflect the degree of inflammation in the disease, but also serve as biomarkers of disease onset and are detected in the patient’s urine (122). As for recurrent FSGS, the mechanism of its occurrence may be related to circulating permeability factors, including soluble urokinase-type plasminogen activator receptor (suPAR), anti-CD40 antibody, cardiolipin-like cytokine 1 (CLCF-1), apolipoprotein A-Ib (ApoA-Ib), calmodulin-dependent serine protein kinases (CASK), microRNAs, and transforming growth factor-β (TGF-β) (119, 123). Recently, CPF (circulating permeability factor)-containing plasma from FSGS patients was found to induce the accumulation of lipid droplets and perilipin-2 expression in podocytes, and perilipin-2 was proposed to be identified as a potential biomarker (124). Whether these circulating permeability factors can be used as specific biomarkers for the diagnosis of FSGS requires further experimental evidence.

#### 3.2.4 IgAN

IgAN is a chronic inflammatory kidney condition caused by the abnormal presence of galactose-deficient immunoglobulin A1 (Gd-IgA1), resulting in the accumulation of immune complexes in the mesangium, ultimately leading to kidney inflammation and damage (125, 126). According to a recent review, the mechanism of IgAN inflammation may be caused by the elevated levels of renal-derived ICs, such as anti-Gd Ig A1 antibodies and/or Ig M antibodies, leading to the deposition of serum Gd-Ig A1, and these antibodies in the mesangial region of IgAN patients (127). Some of the deposits can form the nephritic immune complex (HIT3) and activate the MCs, which leads to an increase in the production of extracellular matrix components, cytokines, and chemokines (128). The entire process is consistent with the multihit hypothesis of IgA nephropathy, including production of galactose-deficient IgA1 (Gd-IgA1; Hit 1), IgG or IgA autoantibodies that recognize Gd-IgA1 (Hit 2), and their subsequent immune complexes formation (Hit 3) and glomerular deposition (Hit 4), which has been widely supported by many studies. Whichever stage of injury occurs triggers glomerular inflammation, which is regulated by the CCL2/CCR axis (129). Since IgAN is defined by immunohistochemical or immunofluorescent detection of glomerular IgA deposits, the diagnosis can only be made by renal biopsy (130). In addition to the predominantly, mainly mesangial cell IgA deposits (sometimes also visible along the capillary wall), complement C3 can be detected, and rarely other complement components (C4d, C1q) and/or (to a lesser extent) IgG. During the treatment of IgAN patients, the extent of disease progression can be monitored by testing for urinary inflammatory biomarkers, thus avoiding further renal biopsies. Soo-Young Yoon et al. detected inflammation-related biomarkers in the urine of IgAN patients by multiplex enzyme-linked immunosorbent assay (ELISA) (131). Compared with normal controls, the IgAN group had higher levels of eight urinary inflammatory biomarkers, including BAFF, MCP-1, CXCL10, GDF-15, IL-6, MBL, TfR, KIM-1, GDF-15 and EGF. Recently, MCP-1 and EGF were proposed as valuable biomarkers of IgAN progression and development of chronic histological lesions (132). Torres et al. demonstrated that the predictive value of the EGF/MCP-1 ratio was significantly higher than that of EGF or MCP-1 alone, histologic grading, creatinine clearance, or proteinuria (132). Ju et al. demonstrated that measurements of urinary EGF improved the prediction of renal outcome (133). Therefore, urinary inflammatory biomarkers can be used as alternative predictive biomarkers in patients with IgAN.

## 4 Pathways in glomerular inflammation

### 4.1 JAK/STAT signaling pathway

After being phosphorylated by Janus kinase (JAK) in the cytoplasm, the signal transducer and activator of transcription (STAT) translocated from the cytoplasm to the nucleus and regulates the expression of relevant genes. This pathway is known as the JAK/STAT signaling pathway (134). Activation of the JAK/STAT signaling pathway can be mediated by various factors such as high glucose (135), advanced glycosylation end products (136), and Adriamycin (137). Studies have shown that these substances can induce inflammation by activating the JAK/STAT signaling pathway at the cellular or animal level (138). Moreover, specific cytokines such as TGF-β (139), rIL-6 (140), and IFN (141), can also activate the JAK/STAT signaling pathway.

Extensive research has been conducted on the role of JAK/STAT in various glomerular diseases, including IgAN (142), DN (143), FSGC (144) and LN (142). The overexpression of STAT has been detected in various renal disease caused by glomerular cell injury, including podocytes in DN mice, human glomerular endothelial cells in an *in vitro* model of LN, and MCs in pediatric FSGS patients (63, 144, 145). Thus, blocking the JAK/STAT signaling pathway is essential for alleviating inflammation in glomerular disease, mainly involving STAT1/3/4.

SOCS, known as suppressor of cytokine signaling (SOCS), is involved in the negative regulation of glomerular disease and act as the negative feedback regulators of JAK/STAT signaling. SOCS-1, a member of the SOCS family, regulates the intensity and length of JAK/STAT signaling by employing various methods such as kinase inhibition and binding to STAT proteins (146, 147). SOCS-1 has been shown to reduce renal damage in diabetic mice by enhancing MCP-1 expression and controlling JAK/STAT phosphorylation (148). The possible mechanism is that the structure of SOCS-1 contains a 12 amino acid N-terminal kinase inhibitory region (KIR), which is essential for inhibition of JAK tyrosine kinase activity. Carlota and her team studied the renal effects of a peptidomimetic peptide in the KIR region of the SOCS-1 structure. They found that the peptide inhibited STAT1/3 activation, lowered the expression of mediators induced by hyperglycemia and inflammatory diseases, and decreased MCs proliferation (149). A recent study indicated that inhibitors of insulin-like growth factor-1 (IGF-1) may improve renal injury by reducing renal inflammation and fibrosis via the SOCS/JAK/STAT pathway (150). MiR-145 is able to inactivate the JAK/STAT signaling pathway by directly targeting colony stimulating factor-1(CSF1), thereby inhibiting apoptosis and inflammatory injury in MCs. This ultimately prevents the progression of LN (143).

IL-35, the newest member of the IL-12 family, is composed of IL-12A (p35) and Epstein-Barr virus-induced (EBI)-3 subunits. These subunits bind to the IL-12Rβ2 and gp130 chains, respectively. The gp130 and IL-12Rβ2 chains can form a heterodimer (IL-12Rβ2:gp130), the IL-35 receptor (151). According to a recent study, IL-35 has been proposed to regulate the JAK/STAT signaling pathway in LN model of MCs (152). IL-35 suppresses angiogenesis and also inhibits the mRNA and protein expression levels of TNF-α and IL-6 through the JAK/STAT1 pathway (153). It is possible that STAT1/4 could form a unique heterodimer and bind to the promoter regions of both IL-35 subunits (p35 and EBI3), leading to the mediation of IL35R signaling (154). In addition, the interaction of IL-35 with IL-35R enhanced the inhibitory signaling of leukocyte-associated immunoglobulin (Ig)-like receptor-1 (LAIR1) on the membranes of MCs, leading to the inhibition of the JAK/STAT signaling pathway (152). IL-35 transduces phosphorylation signaling through the JAK/STAT signaling pathway, which in turn enhances the inhibitory effect of LAIR1, reduces the proliferation of MCs, suppresses massive inflammation, and ultimately inhibits the progression of LN.

PTPN2, a non-receptor protein tyrosine phosphatase, is recognized as a key regulator in controlling metabolism and microinflammation (155, 156). There is growing evidence that PTPN is involved in the development and progression of inflammatory diseases (157, 158), and its main mechanism may be to exert anti-inflammatory and anti-fibrotic effects through inhibition of the downstream mediator STAT3 signaling pathway (159). TC45 is the predominant form of PTPN2 in most species, which shuttles between the nucleus and cytoplasm in response to growth factor and cytokine receptor signaling to dephosphorylate different substrates, including STAT3 (160, 161). In addition, PTPN2 is the only PTP known to regulate STAT1 other than SHP2. TC45 is the major PTP regulator of STAT1, which is hyperphosphorylated and activated in PTPN2-deficient cells (162). It has been shown that knockdown of PTPN2 in mouse intestinal epithelial cells results in severe colitis and leads to increased inflammation and increased cell proliferation through activation of STAT1 (163). Interestingly, there are studies showing that PTPN2 can improve renal lesions and fibrosis in DN by reducing the expression of pro-inflammatory and pro-fibrotic cytokines through inhibiting the STAT1/3 signaling pathway (164).The mechanisms involves PTPN2 preventing kidney injury by inhibiting STAT activation, down-regulating STAT-dependent genes, and inhibiting the proliferation of mouse mesangial cells and endothelial cells. Previous research indicated that the recruitment of PTPN11 (SHP-2) could play a role in transmitting negative signals through LAIR-1 molecules involved in transduction (165, 166). Blocking PTPN11 genetically or pharmacologically result in the inhibition of the JAK2/STAT3 signaling pathway (167). In IL-35-treated lupus mice, it has been suggested that the upregulation of LAIR1 may transmit a negative signal by inhibiting PTPN11 to suppress STAT3 activation in MCs (152). In summary, PTPN may be a potential target for the treatment of inflammation in glomerular diseases, and its mechanism needs to be verified by more experiments.

### 4.2 CCL2/CCR

Chemokines participate in the adaptive immune response by recruiting small cytokines from different cell types, mainly through chemotaxis (168, 169). Inflammatory chemokines such as CCL2, CCL5 (RANTES), and C-X3-C motif chemokine 1 have the ability to induce the migration of leukocytes to injured tissues (170, 171). CCL2, also known as macrophage chemokine-1 (MCP-1), is involved in the development of glomerular diseases by facilitating the recruitment of macrophages via interaction with type 2 C-C chemokine receptor (CCR2) (172). The glomerular expression levels of CCL2 and its receptor CCR2 were found to be elevated in human glomerulopathies (85), and CCL2/CCR2 signaling may mediate the development of a variety of glomerular diseases.

Anja Wilkening et al. discovered that CCL2 secretion was notably elevated in MCs and mural epithelial cells exposed to adriamycin, while there was no significant increase in podocytes and glomerular endothelial cells (85). Adriamycin was also found to induce TNF production in MCs and mural epithelial cells. Moreover, TNF indirectly induced CCL2 secretion in all glomerular cell lines, but did not alter CCR2 expression in these cells. However, CCR2-deficient mice demonstrated lower renal expression of these inflammatory markers (173). Moreover, further evidence suggested that the absence of CCR2 led to decreased macrophage infiltration in the glomerular and tubulointerstitial areas, leading to enhanced renal injury (174–176). The absence of CCR2 did not seem to impact the adriamycin-induced injury to podocytes or glomerular endothelial cells in FSGS mice, indicating that adriamycin might not directly harm these cell types as previously believed (37, 177, 178). Another study also noted that CCL2 can trigger the inflammatory response by binding in a synergistic manner to glycosaminoglycans located in the glomerular endothelial glycocalyx, such as acetyl-heparin sulfate (HS), chondroitin sulfate, and non-sulfated hyaluronic acid (179). When the N-deacetylase/N-sulfotransferase-1 enzyme is specifically knocked out in endothelial cells, there is a notable decrease in glomerular endothelial chemokine and leukocyte binding, resulting in diminished inflammatory responses in experimental anti-glomerular basement membrane nephritis mice (180).

In addition to recruiting leukocytes, CCL2 also recruits other cells to infiltrate the kidney to trigger glomerular inflammation, such as γδ1 T cells. Previous research indicates that the extravasation of γδ1 T cells to target organs is primarily facilitated by CCL2 and involves the expression of CCR2 (174, 181, 182). Whereas high expression of CCL2 has been demonstrated in the MCs of IgAN patients (183). A recent study shows that γδ1 T cells in the peripheral blood of IgAN patients can be recruited to the kidney via the CCL2-CCR2 chemokine axis and enhance CCL2-CCR2 axis-mediated chemotaxis via C5a (184). C5a is a potent chemoattractant of immune cells, released by local complement activation of IgAN leading to C5 cleavage, which leads to renal injury in IgAN by promoting migration of γδ1 T cells. Previous studies have shown that activation of C5a-C5aR1 signaling promotes Th9 cell recruitment and IL-9 levels via CCL20-CCL6, leading to IgAN deterioration (185). In summary, the C5a/CCL2/CCR2 pathway may serve as a potential mechanism to ameliorate glomerular inflammation, primarily by decreasing the recruitment of immune cells.

### 4.3 NLRP3 inflammasome activation

In the innate immune system, inflammatory vesicles are assembled by cell cytoplasm pattern recognition receptors (PRRs) and play a crucial role in responding to pathogen-associated molecular patterns (PAMPs) or danger-associated molecular patterns (DAMPs). Among the five families of PRRs, the NOD-like receptor family, containing the pyrin domain 3 (NLRP3), is the most extensively studied in chronic kidney disease inflammatory vesicles (186). The NLRP3 inflammatory vesicle consists of three proteins: the NLRP3 scaffold, the PYCARD junction protein (ASC), and the active cysteine asparaginase 1 (caspase-1). This structure is crucial for the production of inflammatory factors (187). The NLRP3 protein consists of a C-terminal leucine-rich repeat sequence (LRR), a central nucleotide-binding oligomeric structural domain (NACHT) and an N-terminal pyrin structural domain (PYD). When injury-associated molecules are recognized and bound by the LRR, NACHT will oligomerize, PYD will recruit ASC and pro-Caspase-1 to form NLRP3 inflammatory vesicles, and pro-Caspase-1 will be activated and then cleave pro-IL-1β and pro-IL-18 to produce mature IL-1β and IL-18 (188, 189). Currently, more and more studies confirm the role of NLRP3 in the kidney, including renal fibrosis, oxidative stress, autophagy and pyroptosis, especially glomerular inflammation (189, 190).

Previous studies have shown that the activation of NLRP3 in renal resident cells plays a role in promoting glomerular disease (111, 191, 192). This pathway can be regulated by NF-κB (193). Zhang Lei et al. found that icariin treatment attenuated renal injury in IgAN rats by inhibiting NF-κB-mediated activation of NLRP3 inflammasome (194). Furthermore, research has revealed that NLRP3 can affect the kidney through non-classical pathways. Wenjie Wang et al. demonstrated that NLRP3 enhances TGF-β1 signaling and Smad activation, promoting renal tubulointerstitial inflammation and fibrosis. Interestingly, these effects were found to be independent on inflammatory vesicle components, such as caspase-1 and the cytokines IL-1β and IL-18 (195). Khurrum Shahzad and his team developed a mouse model with podocytes specifically lacking in NLRP3 or caspase-1. They observed that the absence of NLRP3 or caspase-1 prevented hyperglycemia-induced glomerular injury, although the level of protection differed. Mice with podocyte-specific NLRP3 deficiency were fully protected, whereas those with podocyte-specific caspase-1 deficiency only had partial protection (196). This is in line with earlier findings that NLRP3 operates separately from caspase-1 (197).

Evidence has emerged indicating that the crosstalk between autophagy and NLRP3 inflammatory vesicles is significant in various inflammatory diseases and can be mediated by HDAC6 (168). HDAC6, a class IIb deacetylase, plays a crucial role in the regulation of autophagy and the activation of NLRP3 inflammatory vesicles (198, 199). HDAC6 amplifies pro-IL-1β transcription, raises IL-1β release, and worsens inflammation by promoting NF-κB expression and engaging with NF-κB upstream activators (200–202). At the same time, the caspase-1-mediated signaling intermediate Toll-Interleukin-1 Receptor (TIR) structural domain junction induces interferon-beta (TRIF) cleavage, thereby promoting autophagy (203). HDAC6 could be involved in the crosstalk between inflammatory vesicles and autophagy through its regulation of NF-κB. Furthermore, it has been shown that there is a correlation between increased HDAC6 levels and renal dysfunction (204). Whereas treatment with HDAC6 inhibitors can effectively treat LN in NZB/W mice by reducing α-microtubulin acetylation and NF-κB activation in the glomeruli (205). Additionally, P62 is able to stimulate autophagy by suppressing the deacetylase activity of HDAC6, leading to a higher acetylation levels of α-microtubulin or cortical proteins (206, 207). Hence, it is suggested that the regulation of P62 by HDAC6 may indirectly impact α-microtubulin, potentially playing a role in the crosstalk between inflammation and autophagy in glomerular disease. Future studies should investigate this pathway further.

### 4.4 Toll-like receptor signaling

Toll-like receptors (TLR) play an important role in kidney inflammation (208). It is known that some resident renal cells, such as podocytes, MCs, and endothelial cells, also express TLR in response to immune stimulation (209). TLRs belong to the PRR family and consist of 10 isoforms, including TLR1-10. TLR1, 2, 4, 5, and 6 are transmembrane proteins with multiple leucine-rich repeats, serving as the recognition domains for PAMP and DAMP. The TLRs on the surface of these cells are in charge of detecting PAMPs and DAMPs, with the latter triggering inflammation by interacting with TLR2 and/or TLR4 (210). The binding results in TLR dimerization and conformational alterations, causing the recruitment of TIRAP, TRIF, and TRAM adaptor proteins, initiating subsequent inflammatory pathways (88). The TLR signaling cascade results in the translocation of NF-κB and the transcription of pro-inflammatory genes, such as IL-6, IL-1β, and TNF (211, 212).

TLRs have the ability to engage in signaling cascades with PRRs, such as NLRP3, which requires precise coordination to trigger a targeted and efficient immune response (213). Activated TLR4 receptors initiate NF-κB and regulate the production of NLRP3, pro-IL-1β, and pro-IL-18, which in turn induce a variety of pro-inflammatory cytokines involved in diabetes-induced inflammatory response and apoptosis, leading to DN (214, 215). Lei Liu et al. found that miR-181a has the potential to reduce injury in CKD patients by suppressing the CRY1 gene and the TLR/NF-κB pathway (216). TLR4 deficiency results in decreased renal inflammation by blocking NF-κB activation induced by angiotensin II therapy (217). Furthermore, in cultured podocytes, high glucose-induced up-regulation of TLR4 expression can be mediated by ROS production and NF-κB activation (218). High glucose-induced increase in TLR2 and TLR4 expression in monocytes is mediated by activation of the protein kinase C (PKC) pathway (219). Inhibition of toll-like receptor 4-mediated STAT3 activation attenuates angiotensin II-induced renal fibrosis and dysfunction (220). Genipin, a major active ingredient in Gardenia jasminoides, is widely used in traditional Chinese medicine for the treatment of various cardiovascular diseases, where it inhibits Ang II-induced cell proliferation, ROS generation, and pro-inflammatory responses. These effects may be mediated through the TLR signaling pathway (221). Taken together, TLR can occur by interacting with various signaling pathways, and this interaction could potentially be a mechanism for the development of glomerular inflammation.

**Figure 2:**
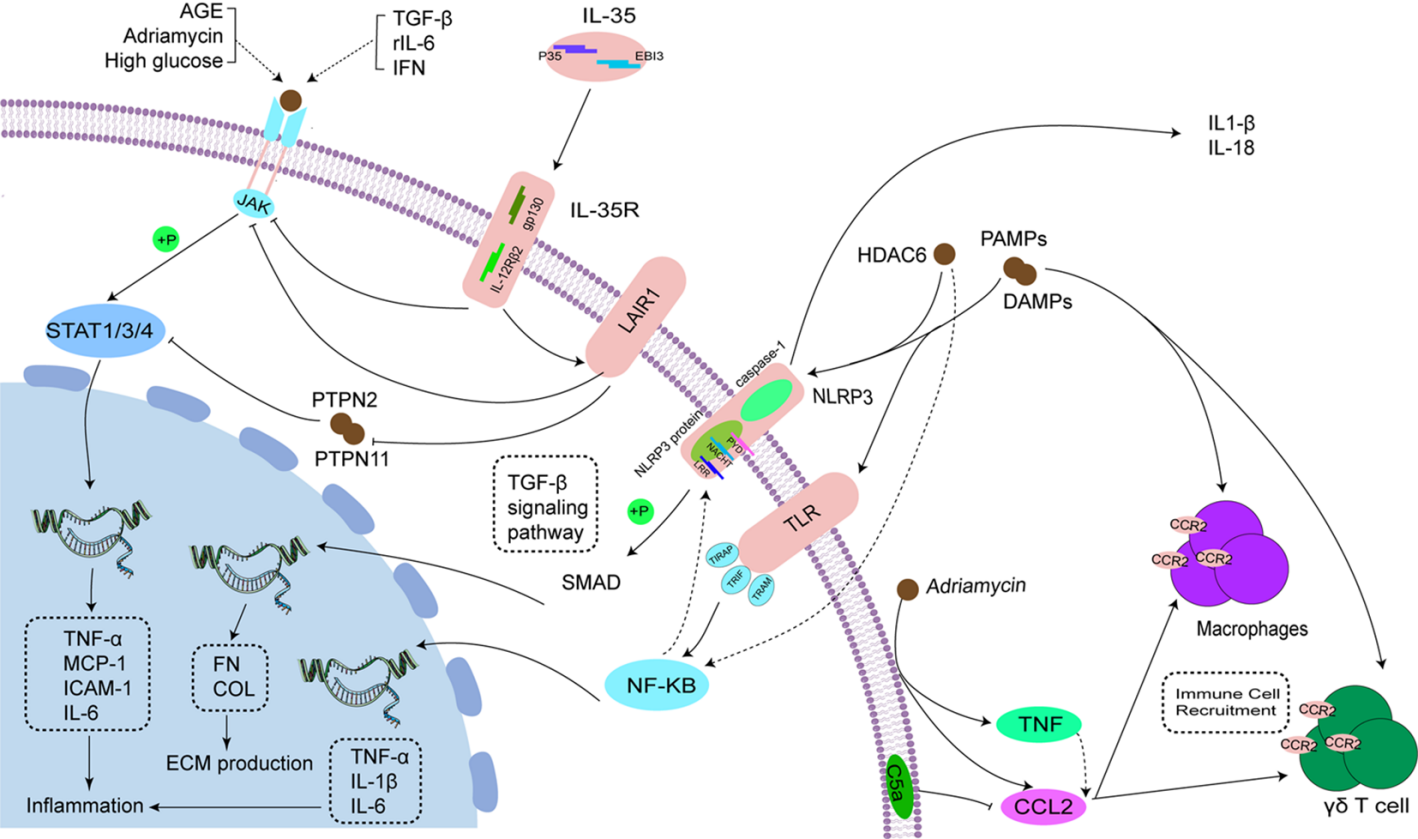
Inflammatory pathways in glomerular disease. This figure depicts the key mechanisms of glomerular disease injury, mainly including the JAK/STAT signaling pathway, CCL2/CCR axis, NLRP3 inflammasome activation, and Toll-like receptor signaling. Several pathological responses, such as oxidative stress, inflammatory response, and fibrosis are among the major causes mediating the activation of these pathways. In addition, several inflammatory factors (including SOCS, IL35, PTPN2, CCL2, NLRP3, etc.) have been found to act on these signaling pathways to modulate glomerular injury.

The pathways associated with glomerular inflammation mentioned above are succinctly summarized in **Figure 2**. The full names of the abbreviations are listed in **Table 2**.

**Table 2:** List of abbreviations.

Abbreviations	Full name in English
GD	Glomerular disease
FSGS	Focal segmental glomerulosclerosis
MCD	Microscopic lesions
IgAN	IgA nephropathy
PMN	Primary membranous nephropathy
MPGN	Membranoproliferative glomerulonephritis
PIGN	Post-infectious glomerulonephritis
MesPGN	Membranoproliferative glomerulonephritis
LN	Lupus nephritis
HIVAN	HIV-associated nephropathy
DN	Diabetic nephropathy
ET-1	Endothelin-1
ICAM1	Intercellular adhesion molecule 1
VCAM1	Vascular cell adhesion protein 1
MCP1	Membrane cofactor protein 1
sICAM-1	Soluble intercellular adhesion molecule-1
AOPPs	Advanced oxidation protein products
JNK	Jun N-terminal kinase
AP-1	Activator of transcription factor 1
GBM	Glomerular basement membrane
GEnC	Glomerular endothelial cells
TNF	Tumor necrosis factor
KLF	Krüppel-like factor
GFR	Glomerular filtration rate
ESRD	End-stage renal disease
NF-κB	Nuclear transcription factors
JAK	Janus kinase
SOCS	Suppressor of cytokine signaling
CSF1	colony stimulating factor-1
EBI	Epstein-Barr virus-induced
LAIR1	leukocyte-associated immunoglobulin (Ig)-like receptor-1
PAMPs	Pathogen-associated molecular patterns
DAMPs	Danger-associated molecular patterns
Caspase-1	Cysteine asparaginase 1
MCs	Mesangial cells

## 5 Prevention and treatment

Glomerular diseases are typically classified according to the histological patterns of kidney injury, with various etiologies and pathophysiologic mechanisms linked to different types of glomerular diseases. The primary challenge in treating these diseases lies in their immune-mediated nature, as well as the diverse clinical manifestations and prognosis they present. Recently, targeted therapies have emerged as more precise treatments for glomerular diseases, that focus on the onset and progression of the disease through the influence of specific molecular or cellular processes. These therapies include biologics and small molecule inhibitors that target inflammatory cytokines, immune cells and signaling kinases (**Table 3**) (222). In a review, Yi-Chan Lin et al. describe relevant targeted therapies for glomerular diseases, including antibody-mediated immune cell exhaustion, complement activation, and signaling (223). However, as we have described, glomerular cells are also involved in the immune response to glomerular disease, and direct targeting of glomerular cells and their signaling is also one of the important options for the treatment of glomerular disease. In recent years, small molecule drugs targeted podocytes have entered clinical trials, offering a glimmer of hope for the treatment of glomerular diseases caused by podocyte injury (224).

**Table 3:** Current clinical trials of novel targeted therapies for glomerular diseases.

Targeted cells	Veterinary drug	Mechanism of action	Glomerular diseases	References
B-cell	Rituximab	Depletes CD20 B cells	PMN, IgAN, FSGS, LN, MCD	NCT00498368
	Telitacicept	Inhibits maturation and activation of B cells	IgAN, LN	NCT04291781
	Atacicept	Inhibits maturation and activation of B cells	IgAN	NCT04716231
	Obinutuzumab	Depletes CD20 B cells	MN	NCT04629248
	Belimumab	Inhibits maturation and activation of B cells	MN	NCT03949855
Podocyte	Atrasentan	ETA inhibitor	FSGS, LN, DN	NCT04573920
	Baricitinib	JAK–STAT inhibitor	FSGS	NCT05237388
	Sparsentan	Dual ETA inhibitor and ARB	IgAN, FSGS	NCT05003986
	Adalimumab	Anti-human TNF antibody	FSGS	NCT04009668
Plasma cell	Felzartamab	Depletes CD38 Plasma cell	MN	NCT04893096

Currently, the therapeutic mechanism of commonly used clinical drugs is mainly to prevent rearrangement of the podocyte and podocyte loss. Angiotensin-converting enzyme (ACE) inhibitors and angiotensin receptor blockers (ARBs) can be utilized to treat Ang II-induced renal disease and enhanced podocyte tensile stress, one of the seminal therapeutic breakthroughs in the history of nephrology (225, 226). Signaling mechanisms reported to cause membrane damage in podocytes include Ang II-mediated Rho-ROCK-related disruption of the actin cytoskeleton and transient receptor potential channel 6 (TRPC6)-mediated release of calcium from intracellular stores (227, 228). TRPC6 is mediated by Ang II (229). As previously described, rearrangement of the foot cytoskeleton is regulated by calcium influx in transient receptor potential channel 6 (TRPC6) as well as by the Rho family. Thus, the use of ACE inhibitors and ARBs to treat Ang II-mediated disruption of podocyte structure and homeostasis provides a mechanistic basis for the treatment of glomerular disease. Similar to ACE inhibitors and ARBs, treatment with SGLT2 inhibitor ameliorated mTORC1-related podocyte injury (230), glomerular inflammation, rearrangement of podocyte cytoskeletal (230), and loss of podocyte (230). The first-line treatment for podocytes is glucocorticoids, supported by a large body of efficacy data and evidence of the immunologic basis of podocytosis. Research indicates that glucocorticoids play a role in stabilizing the actin cytoskeleton and the cleft septal complex (231). Calcineurin inhibitors (CNIs) are suggested as second-line induction and/or maintenance agents for treatment of podocytopathies (232). CNIs have been shown to decrease podocyte damage by stabilizing the actin cytoskeleton, which helps maintain RhoA and tight junction protein ZO-1 (233). In the same way, rituximab treatment of podocytosis led to actin stabilization, preservation of podocyte adhesion, and decrease in apoptosis (234). Kristin Meliambro et al. have also suggested various new therapeutic strategies for targeting podocytes, such as harnessing the regenerative capabilities of podocytes, current clinical trials of drug formulations specific to podocytes, and the creation of drug delivery systems that target podocytes (224). In the future, targeted therapies for podocytes focus more on the above approaches.

## 6 Future prospects

This article reviews studies on the molecular mechanisms, prevention, and treatment of inflammatory responses in various glomerular diseases, especially diabetic nephropathy, lupus nephritis, focal segmental glomerulosclerosis, and IgA nephropathy. The development of glomerular disease as a chronic histologic injury to the kidney is often accompanied by structural and functional damage to glomerular cells, such as endothelial cells, mesangial cells and podocytes. This process does not directly depend on the activation of immune cells, instead, it is mediated through a specific pathway that mainly includes the JAK/STAT signaling pathway, the CCL2/CCR axis, NLRP3 inflammasome activation and Toll-like receptor signaling. We discuss some specific structural injuries in glomerular cells that are associated with the development of glomerular diseases. Pro-inflammatory cytokines expressed by glomerular cells under pathogenic conditions are summarized. This article focuses on a review of the molecular mechanisms, preventive and therapeutic studies of the inflammatory response in different types of glomerular diseases, especially diabetic nephropathy, lupus nephritis, focal segmental glomerulosclerosis and IgA nephropathy. These can help us to understand the signaling pathways that trigger glomerular diseases under pathological conditions and prevent glomerular injury caused by inflammatory responses. In the future, research on the inflammatory response in glomerular diseases should not only focus on immune cells, but also pay more attention to the special structure of glomerular cells and the interactions between cells. This may lead to a better understanding of the molecular mechanisms that trigger glomerular diseases in pathological conditions and aid in preventing glomerular injury from inflammatory responses.
